# Investigating the Possibility of Intervertebral Disc Regeneration Induced by Granulocyte Colony Stimulating Factor-Stimulated Stem Cells in Rats

**DOI:** 10.4061/2011/602089

**Published:** 2010-11-21

**Authors:** Wen-Ching Tzaan, Hsien-Chih Chen

**Affiliations:** ^1^Department of Neurosurgery, Chang Gung Memorial Hospital, 222, Mai-Chin Road, Keelung 204, Taiwan; ^2^School of Medicine, Chang Gung University, 333 TaoYuan, Taiwan

## Abstract

Intervertebral disc (IVD) degeneration is a multifactorial process that is influenced by contributions from genetic predisposition, the aging phenomenon, lifestyle conditions, biomechanical loading and activities, and other health factors (such as diabetes). Attempts to decelerate disc degeneration using various techniques have been reported. However, to date, there has been no proven technique effective for broad clinical application. Granulocyte colony-stimulating factor (GCSF) is a growth factor cytokine that has been shown to enhance the availability of circulating hematopoietic stem cells to the brain and heart as well as their capacity for mobilization of mesenchymal bone marrow stem cells. GCSF also exerts significant increases in circulating neutrophils as well as potent anti-inflammatory effects. In our study, we hypothesize that GCSF can induce bone marrow stem cells differentiation and mobilization to regenerate the degenerated IVD. We found that GCSF had no contribution in disc regeneration or maintenance; however, there were cell proliferation within end plates. The effects of GCSF treatment on end plates might deserve further investigation.

## 1. Introduction

The pathophysiology of disc degeneration is still unknown [1, 2]. Clinically, disc degeneration can be considered as a loss of proper stability and mobility [3, 4]. Morphologically and histopathologically, disc degeneration can be characterized as a decrease in water content associated with proteoglycan reduction of the nucleus pulposus and inner annulus. This effect brings on destruction of annular structure and flattening of the disc [5–7]. In addition, disc tissues have a limited ability to regenerate, since they are avascular and nutritionally supported only by passive diffusion at the end plates [2, 8, 9]. Consequently, once the degenerative process is activated, it is difficult to decelerate and is ultimately considered to be an irreversible condition [2].

One of the most important elements of disc aging and degeneration is the well-recognized decline in the number of disc cells and their products. The experimental reduction of the proteoglycan content in the nucleus is not a new concept; chemonucleolysis studies using the enzyme chymopapain date back over 40 years and were developed as a treatment for disc herniations through a reduction in nucleus pulposus pressure [10]. Negative outcomes from chymopapain led to the introduction of chondroitinase ABC (ChABC) as a new treatment as this enzyme selectively degrades chondroitin-4 sulfate, chondroitin-6 sulfate, and dermatan sulfate glycosaminoglycan chains [11, 12] and is less aggressive than chymopapain. More recently, after the observations of a degeneration-like loss of pressure and disc height [13, 14], ChABC has been specifically used to induce degenerative changes using *in vivo *animal models [15, 16]. Hence, we select this animal model for our experiments.

Administration of granulocyte colony-stimulating factor (GCSF) is known to mobilize hematopoietic stem cells (HSCs) from bone marrow into peripheral blood [17]. Peripheral blood-derived HSCs have been used in place of bone marrow cells in transplantation for the regeneration of nonhematopoietic tissues such as skeletal muscle and heart [18]. GCSF has been used extensively for more than 10 years in the treatment of neutropenia as well as for bone marrow reconstitution and stem cell mobilization [19]; however, it remains unclear whether it is able to stimulates differentiation and mobilization of HSCs into the disc tissues and end plates, and even more, it homes in on intervertebral disc degeneration to promote disc regeneration or at least retard the degeneration.

In this study, we use a rat model to test the hypothesis that GCSF induces HSCs differentiation and mobilization to regenerate the degenerated intervertebral discs. We follow the study up to 6 weeks after experimental induction of disc degeneration and assess the regenerative effects of the GCSF treatment using plain radiography, macroscopic findings, immunohistochemistry, and confocal laser microscopy.

## 2. Materials and Methods

### 2.1. Animal Preparations and Induction of Disc Degeneration

Adult female Sprague-Dawley rats (BioLASCO, Ilan, Taiwan) weighing 260 ~ 320 g were used. Animals were maintained for at least 7 days before the experiment in a temperature-regulated room (23 ~ 25°C) on a 12 h light/dark cycle. Food was withheld but rats were allowed free access to water overnight before surgery. All experimental protocols were in compliance with the NIH *Guide for the Care and Use of Laboratory Animals* and were approved by the Animal Care and Use Committee of the Chang Gung Memorial Hospital.

After 1 week of facility acclimation, rats were operated on using aseptic technique. Rats were anesthetized via intraperitoneal injection of Zoletil 1 mL/kg (Virbac Animal Health) and Rompun 0.5 mL/kg (Bayer HealthCare), respectively. On reaching appropriate depth of anesthesia, animals were placed in a supine position on a heated pad, and an anterior approach to the lumbar spine was performed.

The lumbar spine from L3 to S1 was exposed, and a custom 33-gauge needle attached to a gas-tight microsyringe was inserted through the anterior of the appropriate discs to a controlled depth of 2.5 mm. This insertion depth places the needle tip approximately in the center of the nucleus pulposus. Musculature adjacent to the ChABC and sham PBS injection sites was labeled with a metallic marker for postoperative level identification. The abdominal wall was closed, and animal recovery was monitored for adverse symptoms under heated lamp for 45 minutes. Animals were returned to normal housing and received food and water.

Six weeks after induction of intervertebral disc degeneration, the rats of GCSF treatment group were injected subcutaneously with recombinant human GCSF (50 *μ*g/kg per day) once daily for 5 days.

### 2.2. Bromodeoxyuridine Labeling

Bromodeoxyuridine (BrdU), a thymidine analogue that is incorporated into the DNA of dividing cells during S-phase, was used for mitotic labeling (Sigma Chemical). A cumulative labeling method was used to examine the population of proliferative cells during 5 days of the GCSF treatment. Experimental rats (including 12 normal saline and 12 GCSF treated) were injected intraperitoneally with BrdU (50 mg/kg IP) for 5 consecutive days, starting the day after vehicles (PBS or GCSF) injection.

### 2.3. Tissue Preparation

Immediately after sacrifice, lumbar spines were removed *en bloc *with the ligamentous structures and posterior bony anatomy remaining intact. Spines designated for radiographic analysis were wrapped in PBS-soaked gauze and frozen at −20°C until lateral plain radiographs could be performed. Spines designated for histology were fixed in 10% formalin neutral buffer solution (Wako), decalcified in Plant-Rychlo solution (Decalcifying Solution A, Wako) and dehydrated in a graded series of ethanol (70%, 90%, and 99%, Wako). L2/3 discs with vertebral body units were cut longitudinally at the center of the disc for macroscopic evaluation. L3/4 discs were harvested mainly to make frozen sections for immunostaining, and L4/5 discs were used for proteoglycan evaluation.

### 2.4. Radiographic Analysis

Lateral plain radiographs were taken under anesthesia inhalation in all groups before harvest. Vertebral body heights and disc heights were measured using NIH image software (NIH, freeware at: http://rsb.info.nih.gov/nih-image/). The disc height index (DHI) was calculated for comparisons between groups. Changes in the DHI were expressed as %DHI and normalized to the measured preoperative IVD height (%DHI = postoperative DHI/preoperative DHI × 100).

### 2.5. Immunohistochemistry

To identify the expression of cell type-specific markers in BrdU^+^cells, double immunofluorescence was performed. The rat monoclonal anti-CD34 antibody (CD34, 1 : 200, Acris Antibodies GmbH, Hiddenhausen, Germany) was used. Each transverse section was first treated with primary BrdU antibody conjugated with FITC (1 : 500, Jackson Immunoresearch) staining, followed by treatment with CD34 antibodies with Cy3 (1 : 500, Jackson Immunoresearch) staining. The positive signals were observed by fluorescence microscope. We also used confocal laser scanning microscope to observe BrdU and CD34-double positive cells more clearly.

Proteoglycan accumulation changes in the discs were studied to evaluate disc degeneration. The sections were labeled overnight at 4°C with antirat proteoglycan monoclonal antibody (Chemicon, Temecula, CA, USA), prepared at a dilution of 1 : 200 in PBS. The samples were washed with PBS three times and reacted with antimouse HRP (Dako A/S, Profuktionsvej, Denmark) at a dilution of 1 : 100 in PBS for 30 min at 4°C. Finally, the sections were counterstained with hematoxylin for histologic examination.

### 2.6. Confocal Laser Microscopy

In order to visualize changes in proteoglycan accumulation more clearly, the sections labeled with proteoglycan antibodies were stained with antigoat Alexa fluors 488 (Molecular Probes, Eugene, OR, USA) a fluorescent second antibody and evaluated under confocal laser scanning microscopy. Statistical analyses were performed between the groups by Wilcoxon's paired signed rank test using Stat View software. Significance in all cases were set at *P* < .05.

## 3. Results

### 3.1. Radiographic Analysis

**Figure 1:**
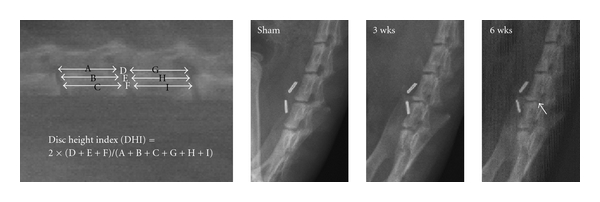
Intervertebral disc height (DHI) was calculated by averaging the measurements obtained from the anterior, middle, and posterior portions of the IVD and dividing that by the average of adjacent vertebral body heights. We found ChABC injection resulted in no significant change at 3 weeks after injection; however, some early osteophyte formation with decrease in DHI was disclosed at 6 weeks after injection.

Using digitized radiographs, measurements including the vertebral body height and IVD height were analyzed. Intervertebral disc height (DHI) was calculated by averaging the measurements obtained from the anterior, middle, and posterior portions of the IVD and dividing that by the average of adjacent vertebral body heights. We found ChABC injection resulted in no significant change at 3 weeks after injection; however, some early osteophyte formation with decrease in DHI was disclosed at 6 weeks after injection (Figure 1). 

### 3.2. Immunohistochemistry

**Figure 2:**
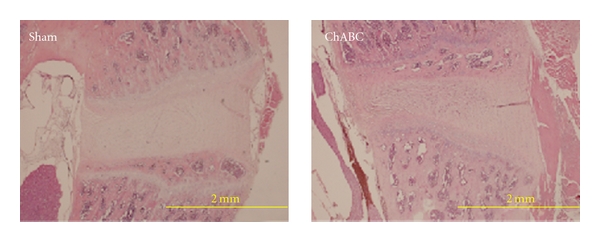
Loosen arrangement with disorganization of nucleus pulposus (NP) was noted after ChABC injection on H & E staining.

**Figure 3:**
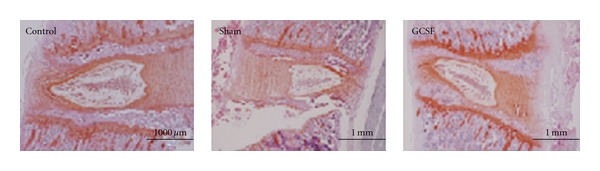
No significant changes of NP after GCSF treatment was noted than sham-operated treatment on the Safranin O staining. However, the GCSF treatment group showed increasing Safranin O staining over end plates even more than normal control group.

Loosened arrangement with disorganization of nucleus pulposus (NP) was noted after ChABC injection on H & E staining (Figure 2). However, there were no significant changes of NP after GCSF treatment than sham-operated treatment on the Safranin O staining. It is worth mentioning that the GCSF treatment group showed increasing Safranin O staining over end plates even more than normal control group (Figure 3). 

### 3.3. Double Immunofluorescence

**Figure 4:**
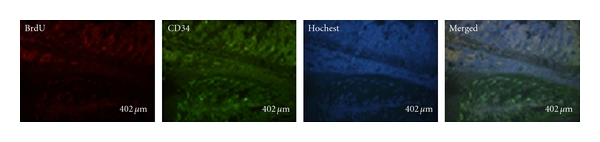
Some positive BrdU staining around the end plates in the GCSF treatment group, however, no apparent CD34 costaining were disclosed.

Although we could find some positive BrdU staining around the end plates in the GCSF treatment group, no apparent CD34 costaining were disclosed (Figure 4).

## 4. Discussion

Histopathologically, the IVD degeneration shows a decrease in water content associated with reduced proteoglycan content of the nucleus pulposus, resulting in destruction of the annular structure and flattening of the disc. Although we had found decrease in DHI since 6 weeks after ChABC injection, the results showed no statistical significance. Probably longer time interval and larger data base are needed for statistic analysis.

Recent advances in molecular biology have provided new knowledge on the nature of the IVD and disc cells. Experimental studies on disc cell function have enabled scientists and clinicians to develop new approaches for the treatment of disc degeneration and regeneration [20]. Currently, strategies to regenerate the disc focus on restoring the ability to regulate matrix production and to restore the disc tissue. In these strategies, autologous NP cell transplantation has become one of the major techniques in attempts to prevent IVD degeneration in animal models [21, 22]. However, it has been considered difficult for broad application clinically. In our study, we hypothesize that GCSF can induce bone marrow stem cells differentiation and mobilization to regenerate the degenerated IVD. We found that GCSF had no contribution in disc regeneration or maintenance; however, there were some increasing staining and cell proliferation within end plates. The effects of GCSF treatment on end plates might deserve further investigation.

## 5. Conclusions

In our study, we found that GCSF had no contribution in disc regeneration or maintenance after induction of disc degeneration for 6 weeks; however, there were cell proliferation within end plates. The effects of GCSF treatment on end plates might deserve further investigation.

## References

[1] Buckwalter JA (1995). Aging and degeneration of the human intervertebral disc. *Spine*.

[2] Nishida K, Kang JD, Gilbertson LG (1999). Modulation of the biologic activity of the rabbit intervertebral disc by gene therapy: an in vivo study of adenovirus-mediated transfer of the human transforming growth factor *β*1 encoding gene. *Spine*.

[3] Frick SL, Hanley EN, Meyer RA, Ramp WK, Chapman TM (1994). Lumbar intervertebral disc transfer: a canine study. *Spine*.

[4] Bao Q-B, McCullen GM, Higham PA, Dumbleton JH, Yuan HA (1996). The artificial disc: theory, design and materials. *Biomaterials*.

[5] Hirsch C, Schajowicz F (1953). Studies on structural changes in the lumbar annulus fibrosus. *Acta Orthopaedica Scandinavica*.

[6] Osti GL, Vernon-Roberts B, Moore R, Fraser RD (1992). Annular tears and disc degeneration in the lumbar spine: a post-mortem study of 135 discs. *Journal of Bone and Joint Surgery*.

[7] Melrose J, Roberts S, Smith S, Menage J, Ghosh P (2002). Increased nerve and blood vessel ingrowth associated with proteoglycan depletion in an ovine anular lesion model of experimental disc degeneration. *Spine*.

[8] Bradford DS, Cooper KM, Oegema TR (1983). Chymopapain, chemonucleolysis, and nucleus pulposus regeneration. *Journal of Bone and Joint Surgery*.

[9] Lipson SJ, Muir H (1981). Proteoglycans in experimental intervertebral disc degeneration. *Spine*.

[10] Saunders EC (1964). Treatment of the canine intervertebral disc syndrome with chymopapain. *Journal of the American Veterinary Medical Association*.

[11] Eurell JAC, Brown MD, Ramos M (1990). The effects of chondroitinase ABC on the rabbit intervertebral disc. A roentgenographic and histologic study. *Clinical Orthopaedics and Related Research*.

[12] Kato F, Iwata H, Mimatsu K, Miura T (1990). Experimental chemonucleolysis with chondroitinase ABC. *Clinical Orthopaedics and Related Research*.

[13] Sasaki M, Takahashi T, Miyahara K, Hirose T (2001). Effects of chondroitinase ABC on intradiscal pressure in sheep: an in vivo study. *Spine*.

[14] Yamada K, Tanabe S, Ueno H (2001). Investigation of the short-term effect of chemonucleolysis with chondroitinase ABC. *Journal of Veterinary Medical Science*.

[15] Norcross JP, Lester GE, Weinhold P, Dahners LE (2003). An in vivo model of degenerative disc disease. *Journal of Orthopaedic Research*.

[16] Hoogendoorn RJ, Wuisman PI, Smit TH, Everts VE, Helder MN (2007). Experimental intervertebral disc degeneration induced by chondroitinase ABC in the goat. *Spine*.

[17] Demetri GD, Griffin JD (1991). Granulocyte colony-stimulating factor and its receptor. *Blood*.

[18] Orlic D, Kajstura J, Chimenti S (2001). Mobilized bone marrow cells repair the infarcted heart, improving function and survival. *Proceedings of the National Academy of Sciences of the United States of America*.

[19] Weaver CH, Buckner CD, Longin K (1993). Syngeneic transplantation with peripheral blood mononuclear cells collected after the administration of recombinant human granulocyte colony-stimulating factor. *Blood*.

[20] Gruber HE, Hanley EN (2003). Recent advances in disc cell biology. *Spine*.

[21] Okuma M, Mochida J, Nishimura K, Sakabe K, Seiki K (2000). Reinsertion of stimulated nucleus pulposus cells retards intervertebral disc degeneration: an in vitro and in vivo experimental study. *Journal of Orthopaedic Research*.

[22] Nomura T, Mochida J, Okuma M, Nishimura K, Sakabe K (2001). Nucleus pulposus allograft retards intervertebral disc degeneration. *Clinical Orthopaedics and Related Research*.

